# A New HPLC-ELSD Method for Simultaneous Determination of N-Acetylglucosamine and N-Acetylgalactosamine in Dairy Foods

**DOI:** 10.1155/2015/892486

**Published:** 2015-12-15

**Authors:** Ho Jin Kim, In Kyung Bae, Min Hee Jeong, Hye Jin Park, Jin Sil Jung, Jang Eok Kim

**Affiliations:** ^1^National Agricultural Products Quality Management Service, Gimcheon 740-871, Republic of Korea; ^2^School of Applied Biosciences, College of Agriculture and Life Sciences, Kyungpook National University, Daegu 702-701, Republic of Korea

## Abstract

A rapid high performance liquid chromatographic method with evaporative light scattering detection (HPLC-ELSD), using a carbohydrate column, was developed for simultaneous determination of N-acetylglucosamine (GlcNAc) and N-acetylgalactosamine (GalNAc) in dairy foods. Sample preparation was performed by precipitation using acetonitrile. The limits of detection were 2.097 mg/L for GlcNAc and 3.247 mg/L for GalNAc. The limits of quantification were 6.043 mg/L for GlcNAc and 9.125 mg/L for GalNAc. Accuracy ranged from 96.4 to 105.7% for GlcNAc and from 97.1 to 104.1% for GalNAc. The precision of the method was <1.7% for GlcNAc and <2.2% for GalNAc. The mean recovery of the method was measured by spiking samples with 30.0–120.0 mg/L GlcNAc or 12.5–50.0 mg/L GalNAc and was found to be 95.1–105.5% for GlcNAc and 99.5–105.9% for GalNAc. The stability test results of standard solutions stored at 4, 20, and 40°C were 96.2–104.7% for GlcNAc and 98.0–106.5% for GalNAc. This study determined GlcNAc and GalNAc in dairy foods using HPLC-ELSD method. This rapid, simultaneous quantitation method might be useful as a mean of convenient quality control of dairy foods.

## 1. Introduction

N-Acetylgalactosamine (GalNAc) is known to be the most potent inhibitor of agglutination of trypsin-treated rabbit erythrocytes, being 6 times more inhibitory than galactose or *α*-linked galactose di- or trisaccharides (melibiose or raffinose) [1]. N-Acetylglucosamine (GlcNAc) has been shown to improve flexibility as well as the condition of cartilage and enhance skin moisturization [2–5]. In a study by Rubin, conducted in 10 people who had problems with flexibility and later consumed 1.5 g poly-GlcNAc every day for 6 weeks, 6 of these individuals demonstrated increased blood concentrations and improved flexibility [6].

GlcNAc and GalNAc are kind of carbohydrates in milk [7, 8]. Free GlcNAc has been found at concentrations ranging from 1.1 to 11 mg/100 mL, [8, 9] and GalNAc has been found at levels from 2.5 to 3.0 mg/100 mL [7]. Milk contains small amount of GlcNAc and GalNAc. For this reason, the development of an accurate method for the detection of extremely small amounts of GlcNAc and GalNAc in dairy foods have become crucial. Recio and Belloque have reported that the content of GlcNAc and GalNAc increased during storage of commercial UHT milk at room temperature [7, 10].

**Figure 1:**
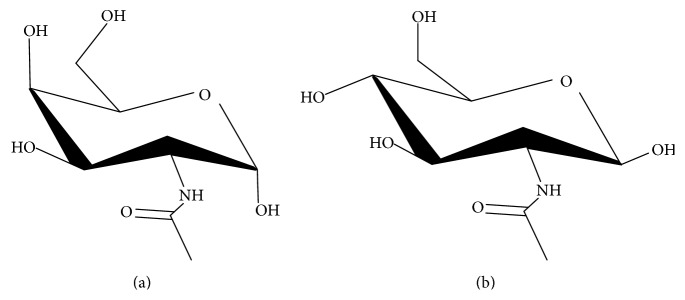
Structure of GalNAc and GlcNAc: (a) GalNAc and (b) GlcNAc.

GalNAc (Figure 1(a), 2-(Acetylamino)-2-deoxy-D-galactose, C_8_H_15_NO_6_, GalNAc) is an amino sugar derivative of galactose. GlcNAc (Figure 1(b), 2-(Acetylamino)-2-deoxy-D-glucose, C_8_H_15_NO_6_, GlcNAc) is monosaccharide derivative of glucose. It is an amide chemical structure between glucosamine and acetic acid. Molecular masses are the same and these structures are closely related making the chromatographic separation difficult. Some technical approaches have been used to measure the contents GalNAc and GlcNAc including gas chromatography (GC) [7]. The major disadvantage of these GC methods consists of sample derivatization. Derivatization has limitations in their applications to sample analyses, such as short detection wavelengths, low detection sensitivity, poor stability, and reducing reproducibility. Therefore, for the determination of GlcNAc and GalNAc other technical approaches were demanded. The analysis of monosaccharides and more complex carbohydrates is often performed by liquid chromatography (LC) techniques, using amino-bonded silica columns [11]. High-performance anion-exchange chromatography with pulsed amperometric detection (HPAEC-PAD) was also developed for analysis of carbohydrates [12, 13]. Many other techniques have been used for the separation of mono- and disaccharides, such as concanavalin A affinity chromatography [14], gas chromatography mass spectrometry (GC/MS) [15, 16], and capillary zone electrophoresis with absorbance [17].

These previous methodologies were not simultaneous analysis for determining GlcNAc and GalNAc. It analyzed fructose, rhamnose, arabinose, galactinol, galactose, and so on which is one part of monosaccharides and more complex carbohydrates. Most of the analysis of carbohydrate studies was performed by complicated derivatization procedure [13, 17]. Moreover in Soria's study evaporation procedure was performed which takes long period on sample preparation [15]. To avoid sample derivatization, a universal mode of detection could be used for evaporative light scattering detection (ELSD).

In recent years, high-performance liquid chromatography- (HPLC-) ELSD has been developed for better quantitation of mono- and oligosaccharides in dairy foods [18]. In addition, ELSD has been widely used to detect nonvolatile compounds, such as carbohydrates, and provides good quantitative results [11, 19, 20]. Moreover HPLC-ELSD system has shown advantages in terms of sensitivity and power resolution in the analysis of complex mixtures of minor carbohydrates [20–22].

The aim of this study was to develop a no derivatization procedure for the simultaneous analysis of dairy foods. The developed method may be employed as a valuable tool for quality control of dairy products analysis of GlcNAc and GalNAc.

## 2. Experimental

### 2.1. Reagents, Solvent, and Standards

Analytical reagent grade methanol (MeOH) and acetonitrile (ACN) were obtained from Fisher scientific (Fair Lawn, NJ, USA). Water was purchased from J. T. Baker (Phillipsburg, NJ, USA). GlcNAc (99%) and GalNAc (99%) standards, Zinc sulfate heptahydrate (99%), and Potassium hexacyanoferrate trihydrate (98.5~102.0%) were purchased from Sigma-Aldrich (St. Louis, MO, USA).

### 2.2. Sample Preparation

Samples (infant formula, yogurt, UHT milk, and raw milk) were immediately frozen and stored at −70°C. Infant formula, yogurt, and UHT milk (Namyang Dairy, Gongju, South Korea) were purchased at a local grocery store in Daejeon city (South Korea). The raw milk samples were collected from Southwestern cattle ranches in South Korea Gongju city. About 1 g of infant formula was weighted and then diluted with 10 mL water. The sample was prewarmed in a 40°C water bath, homogenized by a homogenizer (Omnimacro, model 17505, GA, USA) at 3000 rpm for 5 minutes, and combined with 10 mL ACN. The solution was sonicated at room temperature for 10 minutes and then centrifuged at 13,000 rpm for 10 min at 4°C (Kendro, Hanau, Germany). 1 mL of supernatant was taken and concentrated with N_2_ and then re-dissolved with 1 mL of mobile phase and injected into the chromatographic system. About 2 g of yogurt sample was weighted and then diluted with 2 mL water. The solution was treated using the method described above, infant formula, and then 6 mL of ACN was added. Raw milk and UHT milk were also treated with the same method, but raw milk and UHT milk were not diluted with water. According to the content of total solids in dairy foods, there are a few differences on sample procedures. The crude fat and proteins were precipitated with using Carrez solutions I (2.7 g of Potassium hexacyanoferrate (II) trihydrate in 100 mL water) and II (5.5 g of Zinc sulfate heptahydrate in 100 mL water) and the samples were centrifuged at 13000 rpm for 15 min at 4°C in order to remove fats [13]. The crude fat and protein contents were determined by the method of Association of Official Analytical Chemists [23].

### 2.3. Chromatographic Conditions

The HPLC (Agilent 1100, Palo Alto, CA, USA) equipped with ELSD (Alltech 2000AS, Deerfield, IL, USA) was used to separate and detect GlcNAc and GalNAc. A column, Prevail Carbohydrate ES (250 mm × 4.6 mm, 5 *µ*m) column (Grace Davison Discovery Sciences, Deerfield, IL, USA) was used to separate GlcNAc and GalNAc at 30°C in a column oven. The mobile phase consisted of ACN, water, and MeOH (60 : 20 : 20, v/v) and was pumped at a flow rate of 0.5 mL/min. Isocratic mode was in operation for 30 minutes. The injection volume was 10 *µ*L. The ELSD conditions were as follows: the tube temperature was 85°C and the nitrogen gas flow was 2.0 mL/min.

### 2.4. Method Validation

The validation was conducted according to the International Union of Pure and Applied Chemistry (IUPAC) 2002 guidelines and International Conference on Harmonisation (ICH) 2005 [24, 25].

#### 2.4.1. Calibration Standards and Matrix-Matched Calibration Standards

The stock standard solution of each compound was prepared as follows: an accurately weighed amount of GlcNAc and GalNAc (20 mg and 16 mg, resp.) was placed into a 100 mL volumetric flask and brought up to volume with a mixture of ACN : water : MeOH (60 : 20 : 20, v/v). The final concentration of GlcNAc was 200 *µ*g/mL and the final concentration of GalNAc was 160 *µ*g/mL. These solutions were stored at 4°C. GlcNAc and GalNAc working standard solutions were prepared daily in the range from 25 to 200 *µ*g/mL and 10 to 160 *µ*g/L, respectively, by diluting in mobile phase.

Matrix-matched standard solutions were prepared using raw milk, UHT milk, yogurt, and infant formula. Ranges of 10–200 *µ*g of standards of GlcNAc and GalNAc were weighted and then added to a 100 mL volumetric flask containing the test materials. The final concentrations of GlcNAc and GalNAc were 200 *µ*g/mL and 160 *µ*g/mL, respectively. Matrix stock standard solutions contain the blank test materials with concentration ranges of 25–200 *µ*g/mL for GlcNAc and 10–160 *µ*g/mL for GalNAc.

A calibration curve was used when determining precision, accuracy, recovery, and dynamic range. The standard curve was composed of ten points. We used a third degree polynomial equation of the form *y* = *ax* ^3^ + *bx* ^2^ + *cx* + *d* to define the curve and used the calibration curve to evaluate the concentration of each component.

#### 2.4.2. Selectivity

To evaluate the selectivity of the method, matrix samples of raw milk, UHT milk, yogurt, and infant formula were prepared according to the method (Section 2.3) described above and then were analyzed using the HPLC-ELSD system. Selectivity was investigated by comparing the results obtained for a standard solution of GlcNAc and GalNAc to the same standard solution spiked with four different dairy foods. We attempted to use the ELSD to identify the peaks from GlcNAc and GalNAc in four dairy foods separated using the HPLC-ELSD system.

#### 2.4.3. Precision and Accuracy

Precision studies were carried out by determining the interday and intraday reproducibility of the peak areas. Intraday tests were carried out using four determinations at concentration levels corresponding to 25–150 *µ*g/mL and 10–100 *µ*g/mL of GlcNAc and GalNAc. Precision was based on calculating the relative standard deviation (RSD) of the results for GlcNAc and GalNAc at each concentration. Within-laboratory precision studies were carried out in a single laboratory, with three different operators performing the analysis on four different days using four different dairy matrix foods. Within-laboratory precision was evaluated using one-way analysis of variance (ANOVA) and the *F*-test using SPSS version 12.0. The accuracy of the method was determined based on the percent recovery of GlcNAc and GalNAc amount (10–150 *µ*g/mL) added to the samples.

#### 2.4.4. Limit of Detection (LOD) and Limit of Quantification (LOQ)

The limit of detection (LOD) is defined as the lowest concentration of an analyte in a sample that can be positively identified against background. The limit of quantification (LOQ) is the lowest concentration of an analyte that can be accurately measured with acceptable precision under the operational conditions of the method. The LOD is defined as the concentration when the signal to noise ratio (S/N ratio) is 3. The LOQ is the analyte concentration at which the S/N ratio is equal to 10.

#### 2.4.5. Recovery

Recovery tests were performed by spiking infant formula, yogurt, UHT milk, and raw milk. The concentrations of GlcNAc were 30, 60, and 120 *µ*g/mL, and the concentrations of GalNAc were 12.5, 25, and 50 *µ*g/mL. Recovery was determined by comparing the observed peak area of five replicate dairy foods spiked at each concentration with the expected values at these concentrations as determined from a standard calibration curve.

#### 2.4.6. Stability

To determine the stability of standard solutions samples were stored at 4, 20, and 40°C for 10 days. The individual stock solutions of GlcNAc and GalNAc were prepared in mobile phase and the concentration of 25, 150 mg/L for GlcNAc and 10, 100 mg/L for GalNAc was diluted with the same solvent composition. In order to gain stability of samples, infant formula, yogurt, UHT milk, and raw milk also were stored at 4, 20, and 40°C for 10 days. Samples were performed in triplicate for three-temperature level to determine stability. The standard solution and samples stored at 4°C were kept in a refrigerator, and the samples stored at 20 and 40°C were stored in a microorganism incubator (Tuttlingen, BINDER GmbH, Germany).

## 3. Result and Discussion

### 3.1. Sample Treatment

GlcNAc and GalNAc were extracted from the dairy foods, using ACN or Carrez solution. Highest resolution was achieved using extraction by ACN. The crude fat content was 2.0 mg/100 mL using Carrez solution and 12.7 mg/100 mL using ACN solution. The crude protein content was 9.1 mg/100 mL using Carrez solution and 24.5 mg/100 mL using ACN solution. The ACN extracted solution was reduced crude fat and proteins in dairy foods (infant formula, yogurt, UHT milk, and raw milk). In the case of crude fat and protein, the Carrez method showed the lowest content of crude fat. However, when samples were extracted via the Carrez method, it was found that GalNAc remained unseparated and the sensitivity to GlcNAc was low. In the case of extraction with ACN, the ratio of elimination in crude fat and crude protein was lower than that of the Carrez method but the peaks resolved the most clearly with ACN method and showed high yielding results for GlcNAc and GalNAc. The yields obtained in the extraction step for Carrez solution and ACN were 67.2~75.3% and 95.1~105.9%, respectively. Moreover, the ACN method was very easy to utilize for sample handling and accurate analysis was possible. Therefore, ACN was selected as the extraction solvent.

### 3.2. Method Validation

#### 3.2.1. Calibration Standards and Matrix-Matched Calibration Standards

Calibration curve for GlcNAc and GalNAc was evaluated using ten-pointed calibration on four dairy foods (infant formula, yogurt, UHT milk, and raw milk). The determination coefficient (*r* ^2^) was >0.999 for each standard curve. Calibration curves obtained by using mobile phase solutions covered the concentration range of 25–200 mg/L for GlcNAc and 10–160 mg/L for GalNAc. The response function of the ELSD is known to be nonlinear [26]. The response of the ELSD was best described by a third degree polynomial equation. The correlation coefficient of third degree polynomial equation was >0.999.  Table 1 is shows the parameter of regression equation of GlcNAc and GalNAc. Aliquots of 10 *µ*L of each standard solution were used for HPLC-ELSD analysis. The standard solution injections were performed in triplicate for each concentration level. Excellent third degree polynomial correlation was observed for the analytes between peak areas and concentrations over the range tested. This equation was used to determine two-compound content in dairy foods.

Table 2 compares results obtained for four test materials fortified with three different levels of GlcNAc and GalNAc as determined using either matrix calibration or calibration obtained from standards dissolved in mobile phase solution. The detailed data are given in Table 2. In most cases, the results obtained using calibration solution prepared in the same solvent as the mobile phase were more similar to the true values than those calculated using a calibration prepared from matrix-matched standards.

#### 3.2.2. Selectivity

**Figure 2:**
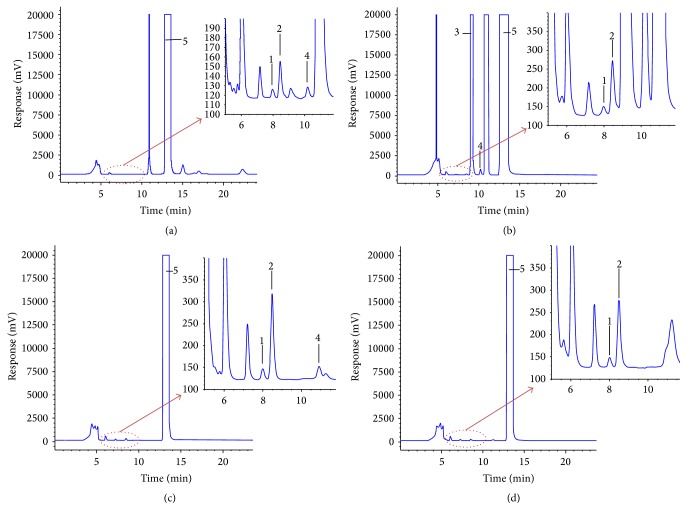
HPLC-ELSD chromatograms of undiluted samples of (a) infant formula, (b) yogurt, (c) raw milk, and (d) UHT milk. Experimental conditions as in Section 2.3 (1: GlcNAc, 2: GalNAc, 3: fructose, 4: sucrose, and 5: lactose).

The mixture of GlcNAc and GalNAc was successfully separated by the optimum chromatographic conditions (Section 2.3).  Figure 2 shows typical chromatograms of four dairy foods from infant formula, yogurt, raw milk, and UHT milk (Figures 2(a)–2(d), resp.), obtained with the HPLC-ELSD system under optimized HPLC-ELSD conditions on a carbohydrate column. The ACN : water : MeOH ratio was 60 : 20 : 20 (v/v), the flow rate was 0.5 mL/min, and the column temperature was 30°C. No less than five replicate samples were examined for each dairy food. Chromatograms of GlcNAc and GalNAc showed no matrix interferences from raw milk, UHT milk, yogurt, or infant formula. Stable retention times and optimal resolution were achieved using a mobile phase composed of ACN, water, and MeOH in the ratio of 60 : 20 : 20 (v/v). These results which are identification of peaks from GlcNAc and GalNAc showed satisfactory selectivity on HPLC-ELSD system.

#### 3.2.3. Precision and Accuracy

The precision of the chromatographic system was tested by performing five independent intra- and interday replicate measurements of a standard solution containing GlcNAc and GalNAc and then checking the RSD of the peak areas. Five independent replicates were performed each day for five consecutive days.  Table 3 showed the intraday and interday RSD values for the peak areas. The RSD values for intraday precision were <2.2% for both GlcNAc and GalNAc. The intraday accuracy was 96.4–105.7%. Interday values were, in most cases, less than 1.8%, independent of the concentration level. The interday accuracy was 99.3–103.7%. At a 5% significance level, the one-way ANOVA test indicated that there were no significant differences between the three operators. Therefore, it can be concluded that the methods were similar regardless of the operator or day of analysis. The detailed data is shown in Table 4. The low intra- and interday RSD and accuracy values indicate robust validation of the method.

#### 3.2.4. Limit of Detection (LOD) and Limit of Quantification (LOQ)

LOD and LOQ under the present chromatographic condition were determined on the basis of response and slope of each regression equation at a signal to noise ratio (S/N) of 3 and 10, respectively. Matrix spiked method was used for LOD and LOQ calculation. The LOD and LOQ ranged from 2.097 to 6.043 *µ*g for GlcNAc and from 3.247 to 9.125 *µ*g for GalNAc, respectively.

#### 3.2.5. Recovery

The recovery rates were close to 100% in almost all cases. The recovery of the method was satisfactory with accuracy ranging from 95.12% to 105.88%, respectively. Considering the results of the recovery test, this method can be considered accurate. The detailed data is shown in Table 5.

Endogenous concentration of the GlcNAc and GalNAc from dairy foods were determined by constructing a five-point calibration curve using HPLC-ELSD conditions identical to those used for the test materials. Analyte concentrations were calculated according to the following:

(1) $$\begin{array}{c} mg/L=C_{1}\times \frac{V_{1}}{V_{2}}, \end{array}$$

where *C*_1_ is concentration of analyte determined from the standard calibration curve, *V*_1_ is total volume of extraction solution, and *V*_2_ is sample weight.

The contents of GlcNAc and GalNAc found in dairy foods are shown in Table 5. During analysis, the raw milk sample was found to contain 152.3 mg/L of GlcNAc and 189.3 mg/L of GalNAc. We found a larger amount of GalNAc than was reported by J. Belloque paper, but GlcNAc content was similar [7].

#### 3.2.6. Stability

The stability of standard solution was tested at 4, 20, and 40°C for 10 days. The stability of standard solution was satisfactory with accuracy ranging from 96.20 to 106.50%. We conclude that standard solutions are stable for at least 10 days at three different temperatures. The detailed data is shown in Table 6. We tested the samples that contain raw milk, UHT milk, yogurt, and infant formula at 4, 20, and 40°C for 10 days. According to Belloque et al. [7], the amount of N-acetylglucosamine increases over time. However, the experimental results of the storage stability over 10 days were determined and indicated as safe (Table 6).

## 4. Conclusions

In this paper we report a simple, rapid, and sensitive HPLC-ELSD method for the simultaneous quantification of GlcNAc and GalNAc in dairy foods. The developed method could be employed as a valuable tool during routine analysis for GlcNAc and GalNAc in dairy foods, as the ability to simultaneously determine the levels of these components in dairy foods will play an important role in research.

Validation experiments were performed using assays with standard solutions, samples, and spiked samples to evaluate the quality of the data and ensure the reliability of the method. Method linearity, accuracy, precision, stability, and selectivity were evaluated, as were limits of detection and quantification. The simultaneous method described here offers a convenient means for quality control of dairy foods.

## Figures and Tables

**Table 1:** Parameters of regression equations for calibration curves and range.

Compound	*a*	*b*	*c*	*d*	*R* ^2^ ^a^	Range (*μ*g/mL)
GlcNAc	−7.353*e* ^−4^	4.489*e* ^−1^	12.327	79.609	0.9999	25–200
GalNAc	−1.922*e* ^−3^	6.089*e* ^−1^	−4.936	252.349	0.9997	10–160

For each curve the equation is *y* = *ax* ^3^ + *bx* ^2^ + *cx* + *d*, where *y* is the peak area and *x* is the concentration of the analyte (*μ*g/mL).

*a* is the third degree constant.

*b* is the second degree constant.

*c* is the first degree constant.

*d* is the intercept.

*R* ^2^ ^a^ = the correlation coefficient.

**Table 2:** Comparison of results obtained by standard solution-based and matrix-matched calibration.

Matrix	FC^c^	Recovery (%)			
		GlcNAc		GalNAc	
		S^a^	M^b^	S^a^	M^b^
Raw milk	25	101	100	98	101
	50	101	97	97	100
	100	102	100	99	102

UHT milk	25	98	100	100	100
	50	99	102	101	101
	100	102	101	99	100

Yogurt	25	103	103	98	97
	50	99	99	99	98
	100	99	101	100	99

Infant formula	25	99	100	100	100
	50	103	102	101	103
	100	102	102	102	102

^a^S = standard solution calibration.

^b^M = matrix-matched calibration.

^c^FC = fortified concentration (mg/mL).

**Table 3:** Intra- and interday precision and accuracy data of GlcNAc and GalNAc determination (*n* = 5 independent replicates).

Compound	FC^a^	Intraday			Interday		
		MAC^b^	RSD^c^	AC^d^	MAC^b^	RSD^c^	AC^d^
GlcNAc	25	24.11	1.2	96.4	25.10	1.7	100.4
	75	75.10	0.4	100.1	74.49	1.6	99.3
	120	121.78	0.7	101.5	121.68	1.0	101.4
	150	158.48	0.3	105.7	153.17	1.3	102.1

GalNAc	10	10.31	2.2	103.1	10.37	1.7	103.7
	25	24.55	2.0	98.2	24.91	1.8	99.6
	50	48.54	1.7	97.1	50.13	1.6	100.3
	100	104.07	0.7	104.1	101.95	1.1	101.9

^a^FC = fortified concentration (mg/mL).

^b^MAC = mean absolute concentration (mg/mL).

^c^RSD = relative standard deviation (%).

^d^AC = accuracy of GlcNAc and GalNAc (%).

**Table 4:** Within-laboratory precision, expressed as relative standard deviation for different working days (RSD), for four different matrices fortified with GlcNAc and GalNAc at the same concentration as was originally detected (*n* = 5 independent replicates, obtained on three different days by different analysts).

Recovery (%)								
Operator	Raw milk		UHT milk		Yogurt		Infant formula	
	Recovery	RSD^c^	Recovery	RSD^c^	Recovery	RSD^c^	Recovery	RSD^c^
GlcNAc								
A^a^	101.7	0.5	101.7	0.7	102.1	0.8	97.7	1.1
B^a^	101.8	1.0	101.6	0.1	98.6	1.2	100.9	0.5
C^a^	102.4	0.6	103.8	0.5	100.0	1.0	102.0	1.0

GalNAc								
A^b^	102.5	12	98.5	0.2	101.6	1.2	101.8	2.3
B^b^	101.8	0.1	101.2	0.7	101.0	0.5	100.0	0.5
C^b^	98.4	1.3	99.4	1.3	101.2	0.4	101.3	0.5

^a^The analyses were performed for determination of GlcNAc by three different operators using four different matrix samples in different days.

^b^The analyses were performed for determination of GalNAc by three different operators using four different matrix samples in different days.

^c^Relative standard deviation (%).

**Table 5:** Recovery and endogenous concentration data of GlcNAc and GalNAc from four different dairy foods (*n* = 5 independent replicates).

Matrix	GlcNAc				GalNAc			
	EC^a^	FC^b^	Recovery (%)	RSD^c^	EC^a^	FC^b^	Recovery (%)	RSD^c^
Raw milk	152.3 ± 3.4	30.0	102.06	1.94	189.3 ± 12.1	12.5	100.50	2.46
		60.0	101.41	1.18		25.0	99.92	1.37
		120.0	95.12	0.59		50.0	103.30	2.08

UHT milk	150.2 ± 5.4	30.0	102.39	3.21	115.0 ± 1.1	12.5	103.34	1.81
		60.0	97.02	0.42		25.0	105.88	0.84
		120.0	101.06	2.99		50.0	102.70	0.57

Yoghurt	151.2 ± 11.1	30.0	101.20	0.66	157.0 ± 5.5	12.5	99.39	0.91
		60.0	100.20	0.79		25.0	100.58	0.92
		120.0	100.81	1.03		50.0	99.79	0.62

Infant formula	720.0 ± 1.6	30.0	105.45	0.68	1118.4 ± 12.3	12.5	100.56	1.38
		60.0	103.95	2.90		25.0	102.17	0.81
		120.0	103.26	1.25		50.0	99.51	1.10

^a^EC = endogenous concentration (mg/L, mg/kg).

^b^FC = fortified concentration (mg/mL).

^c^RSD = relative standard deviation.

**Table 6:** Stability results for GlcNAc and GalNAc standard solutions after ten days at 4, 20, and 40°C (*n* = 5 independent replicates).

Compound	ST^a^	FC^b^	Stability (*n* = 5, mean) (°C)									
			Day 1		Day 2		Day 3		Day 7		Day 10	
			AS^c^	RSD^d^	AS^c^	RSD^d^	AS^c^	RSD^d^	AS^c^	RSD^d^	AS^c^	RSD^d^
GlcNAc	4	25	100.16	0.04	98.32	0.28	102.16	0.77	104.72	0.52	104.20	1.54
		150	99.72	1.04	101.84	0.27	101.59	1.25	100.33	0.28	102.00	0.80
	20	25	98.32	3.32	98.76	3.60	99.64	2.92	102.64	1.85	96.20	3.99
		150	99.72	1.11	99.69	0.83	101.79	0.79	101.49	1.29	102.21	0.75
	40	25	102.36	3.27	103.44	2.65	101.72	3.73	104.56	0.66	104.16	1.74
		150	101.99	1.12	102.88	0.46	102.30	0.15	101.84	0.43	97.99	1.34

GalNAc	4	10	103.50	1.81	102.50	1.23	103.70	2.79	105.70	0.20	103.10	4.64
		100	98.58	1.11	98.54	0.42	105.04	0.38	104.86	0.53	101.25	0.49
	20	10	98.00	1.75	103.40	3.95	105.08	3.54	102.50	1.27	105.70	3.63
		100	102.36	1.05	103.53	1.08	103.05	0.78	104.24	1.14	98.68	0.37
	40	10	99.00	1.08	106.50	3.83	102.10	4.98	100.90	3.84	98.40	4.37
		100	101.58	0.21	100.77	2.16	101.66	3.34	100.24	2.96	100.46	0.17

^a^ST = storage temperature (°C).

^b^FC = fortified concentration (mg/L).

^c^AS = accuracy of stability test for GlcNAc and GalNAc (%).

^d^RSD = relative standard deviation (%).
